# On-Line Remaining Useful Life Estimation of Power Connectors Focused on Predictive Maintenance

**DOI:** 10.3390/s21113739

**Published:** 2021-05-27

**Authors:** Jordi-Roger Riba, Álvaro Gómez-Pau, Jimmy Martínez, Manuel Moreno-Eguilaz

**Affiliations:** 1 Electrical Engineering Department, Universitat Politècnica de Catalunya, 08222 Terrassa, Spain; jimmy.arturo.martinez@upc.edu; 2 Electronics Engineering Department, Universitat Politècnica de Catalunya, 08222 Terrassa, Spain; alvaro.gomez-pau@upc.edu (Á.G.-P.); manuel.moreno.eguilaz@upc.edu (M.M.-E.)

**Keywords:** electrical connector, contact resistance, remaining useful life, reliability, data acquisition, maintenance, parameter identification

## Abstract

Connections are critical elements in power systems, exhibiting higher failure probability. Power connectors are considered secondary simple devices in power systems despite their key role, since a failure in one such element can lead to major issues. Thus, it is of vital interest to develop predictive maintenance approaches to minimize these issues. This paper proposes an on-line method to determine the remaining useful life (RUL) of power connectors. It is based on a simple and accurate model of the degradation with time of the electrical resistance of the connector, which only has two parameters, whose values are identified from on-line acquired data (voltage drop across the connector, electric current and temperature). The accuracy of the model presented in this paper is compared with the widely applied autoregressive integrated moving average model (ARIMA), showing enhanced performance. Next, a criterion to determine the RUL is proposed, which is based on the inflection point of the expression describing the electrical resistance degradation. This strategy allows determination of when the connector must be replaced, thus easing predictive maintenance tasks. Experimental results from seven connectors show the potential and viability of the suggested method, which can be applied to many other devices.

## 1. Introduction

Power connectors are critical components, playing a key role in the correct and stable operation of power systems. After long-time operation, degradation mechanisms can negatively impact the contact resistance, and thus performance. Severe outages generated because of power connector failure may lead to costly and catastrophic consequences [1].

Medium voltage connectors are copper or aluminum devices designed to provide a low and stable electrical connection between two conductors or bus bars. These electromechanical devices aim to transmit electrical power with minimum power losses [2,3], thus with minimum voltage drop. The most common materials for medium voltage connectors are copper and aluminum [4] and they are often of compressed type, since compression provides a reduced contact resistance and a reliable electrical connection.

The electrical resistance has two main terms, i.e., the bulk resistance given by its geometry and the resistivity of its constitutive materials, and the contact resistance, which depends on diverse factors such as the pressure of the contact interface [5], the state and roughness of the mating interfaces, the presence of dirt and debris, etc. Connector degradation is often a consequence of an increase in the electrical resistance, which raises its operating temperature. As a result, overheating and consequently a reduction in useful life are expected [6]. Contact resistance increases as a result of a poor contact between the conductor and connector due to different causes, such as deficient installation or temperature cycling produced by daily demand cycles, with consequent expansion and contraction patterns which lose the contact. When the connector resistance is above a certain value, it must be substituted to prevent any failure.

Health management and prognostics allows assessment of the reliability of different elements in their life cycles, while mitigating risks of sudden breakdowns [7]. Today, health management and prognostics is evolving from failure management to degradation management [8]. Reliability engineering is directly related to predicting the remaining useful life of systems by incorporating all available data [9]. The commercialization of low-cost sensors and the development of the Internet of Things (IoT) are facilitating the development of RUL strategies.

The remaining useful life of a power connector is the expected operating time between the present time and the time when it needs to be replaced [10]. Correct RUL prediction of power connectors is of great interest to ensure satisfactory reliability and safety of power systems, which provides reference data to develop maintenance plans and schedules, while allowing optimization of power system operational efficiency, to reduce costs due to unscheduled failures and to avoid premature connector faults and major power system failures [11]. Reliability analysis has been typically based on predicting failure rate [12]. Power connectors are designed for a continuous operation of several years, in the order of 30–40 years for substation connectors, although premature failure modes may happen significantly before [13]. Accurate RUL estimation is appealing in order to apply predictive maintenance programs, since this allows determination of and planning for the connectors which must be replaced in the short or medium term, thus avoiding their failure, as well as the undesired consequences of these catastrophic faults, while enhancing assessment of their operating condition [14].

Predictive maintenance is evolving towards data-driven methods and physical modeling due to the widespread use of different types of sensors, communication protocols and computation systems. Grid reliability can be improved by means of online sensing technologies based on low-cost sensors [15]. Regardless of the progress in predictive maintenance methods, hands-on and time-based maintenance are still intensively applied [16].

In the case of devices in which the natural ageing under regular operating conditions requires a long time, accelerated ageing methods are widely applied to analyze their long-term performance [17]. However, this strategy is very expensive in terms of energy consumed, human labor, required materials and monetary cost, and the attained results are specific, thus often lacking generalization capability. Therefore, due to the above mentioned issues, this work avoids the application of this strategy. In addition, conventional maintenance strategies are based on failure data of similar devices; however, such strategies do not consider the specificity of the degradation process of each single device [18]. The approach presented in this paper overcomes this limitation since the predicted RUL is adjusted to each particular connector.

This work develops a low-cost method for on-line monitoring of the electrical resistance of the connector and its evolution over time for a reliable on-line estimation of the remaining useful life based on voltage drop, current and temperature measurements, focused on improving and facilitating predictive maintenance plans.

The method proposed in this paper to determine the RUL is based on acquiring on-line data (voltage drop across the connector, electric current and temperature) to determine the current value and the evolution of the electrical resistance of the analyzed connector, since this is a suitable indicator of the health status of the connector [19], thus allowing determination of its power efficiency and expected lifetime [20]. Any increase in the resistance implies a rise in the temperature of the connector, which further raises the resistance, thus tending to deteriorate the connector performance and reducing the RUL [13]. Next, from a mathematical RUL model, the right moment to replace the connector is calculated, i.e., when the connector will show poor or degraded electrical performance [4]. The RUL model proposed in this work is based on the oxidation multi-spot equation which describes the rise of the electrical contact resistance with time, due to the growth of oxide films at the contact interface [18]. This on-line method allows the prediction of and planning for the moment to replace the connector, based on determining its RUL.

**Figure 1:**
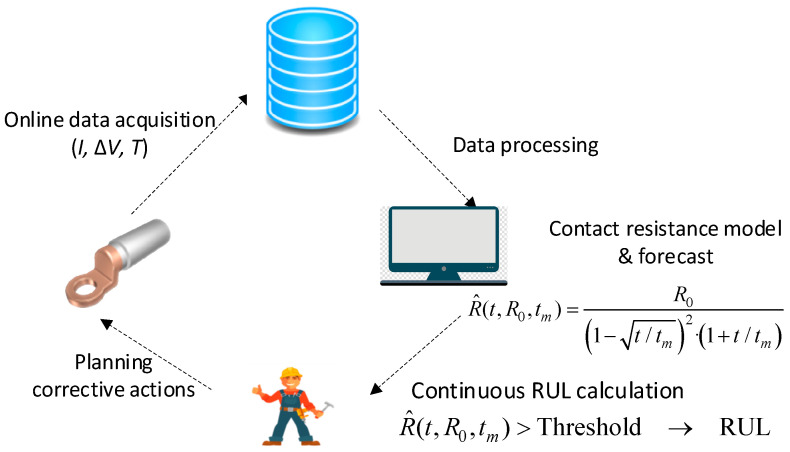
Approach applied to predict the RUL of power connectors.

Figure 1 summarizes the strategy proposed in this work to determine the RUL, which is based on measuring on-line the electrical resistance from the connector temperature, the voltage drop and the current flowing across the connector, and the model of degradation of the electrical resistance from which the RUL is determined. based on when the electrical resistance surpasses a predefined threshold value.

Electrical connectors’ reliability has been often characterized by the failure rate in spite of the misleading results and limitations of this approach [12]. Therefore, remaining useful life (RUL) prediction models are required, although there is a scarcity of literature for power connectors. In [21], the joint effect of vibration and temperature stresses on the value of the contact resistance of automotive connectors was evaluated with the aim of determining the minimum vibration amplitude for fretting-corrosion degradation. In [22], the mechanical behavior and fatigue lifetime of micro electrical connectors were estimated based on the effect of fretting wear. In [12], a RUL method for aviation connectors is presented based on high vibration stress, which combines a vibration-induced physical model, a particle filtering data-driven approach and accelerated degradation testing to evaluate the performance of the proposed approach. In [23], the reliability and failure rate of electrical connectors was predicted under particulate contamination and temperature stresses by means of accelerated tests to collect degradation data. A similar approach was applied in [24], where in this case the lifetime distribution was described by a two-parameter Weibull distribution, whose parameters were inferred by applying the maximum-likelihood estimation method from degradation test data. RUL estimation of micro switches based on Bayesian updating and expectation maximization combined with strong tracking filtering was presented in [25], the failure threshold being based on the contact voltage drop between contacts once closed, which is directly related to the contact resistance. In [26], failure indicators based on the change of the electrical resistance of small size socket electrical connectors are applied to predict the RUL of such components based on random vibration tests carried out to produce fretting corrosion degradation. Resistance was measured by applying the resistance spectroscopy technique jointly with phase sensitive detection, and a Kalman filter was applied for estimating the health status of the socket connector.

This paper proposes a novel RUL estimation method and a criterion for power connectors based on on-line monitoring of the electrical resistance of the connector. This approach is focused on easing predictive maintenance plans, thus contributing to this area due to the scarcity of works in the field of RUL estimation methods for power connectors. The proposed criterion has been experimentally assessed by using a contact resistance based degradation model [27], showing promising results. The strategy proposed in this work to determine the RUL of power connectors is novel, since it is based on on-line measurement of the electrical resistance using low-cost sensors, and on a simple and fast-to-calculate aging model of electrical resistance based on only two parameters which are identified by applying a generalized least squares fitting based on the Nelder-Mead gradient-free minimization algorithm. Therefore, this process is quite simple, with low mathematical complexity and, in consequence, with low computational burden, a fact which makes it feasible to be embedded in the field via Internet of Things (IoT) devices or wireless sensor networks. Another advantage of this proposal is that it does not require the performance of earlier accelerated ageing or degradation tests as in [28], which are time-consuming, expensive and consume high amounts of energy, since it has the ability to continuously update the model from the newly acquired data. It is worth noting that a similar approach can be applied to many other power devices.

## 2. Resistance-Based Degradation Models

### 2.1. Connector’s Electrical Resistance

This section describes the method used for on-line monitoring of the electrical resistance of the connectors, which, as explained, is a good health status indicator, although its value depends on its instantaneous temperature value. However, this measurement supposes a challenging task due to the low values of the connectors’ resistance, which is in the order of some tens of micro-Ohms.

Connectors are supplied at power frequency, so by measuring on-line the voltage drop between the connector terminals Δ*V_Connector_* and the AC current *I* passing through them, the impedance of the connector *Z_Connector_* is determined instead of the resistance *R_Connector_* [4],

(1) $$R_{Connector}=Z_{Connector}cos\varphi =(\Delta V_{Connector}/I)cos\varphi$$

*φ* being the phase angle between the voltage drop across the connector and the current waveforms.

Since temperature has a great impact on the connector’s resistance, temperature is always corrected to 20 °C to remove or at least to minimize the effect of temperature. This correction is applied as [29,30],

(2) $$R_{Connector,\;20{}^{\circ}C}=\frac{\Delta V_{Connector}/I}{1+\alpha_{R}\left(T-20\right)}cos\varphi$$

where *R_Connector_*_,20__°C_ is the resistance converted to 20 °C reference, and α*_R_*, is the temperature coefficient, which is 0.004 K^−1^ for both copper and aluminum. 

It is worth noting that although the temperature can also indicate possible failure modes in the connector, other variables including the electrical current flowing through the connector, vibrations [31], the ambient temperature or different meteorological variables such as wind speed, rain or ice, may produce appreciable changes in the contact resistance. Although the temperature is a key factor affecting the thermal loss of life [32], to use the temperature to indicate the health status, a complete thermal model of the connector is usually required. Although this process is possible to develop, it is much more complex than the approach proposed in this paper due to the inherent complexity of the thermal models, the need to be experimentally validated under different meteorological conditions (wind speed and direction, fog, rain, ice, etc.) and the fact that the thermal model must be adjusted to each type of connector.

### 2.2. Resistance Degradation Model

This section details the contact resistance degradation model used to predict the RUL of the connectors, which is a requirement in order to forecast the time-evolution of the resistance.

The resistance degradation model analyzed in this paper is based on the increase of the contact resistance with time. According to [27], the contact lifetime is inversely dependent on the diffusion coefficient of the oxidizing agent at the contact interface. It is known that time increases the growth of oxide films at the contact interface under fretting conditions [27], thus increasing the electrical resistance, this effect being boosted with the presence of higher temperatures, which in turn increases the effective resistance and the rate of diffusion, thus promoting a faster growth of oxide films at the contact interface.

According to [27], the two-parameter (*R*_0_,*t_m_*) degradation model of electrical resistance describing the time evolution of the contact resistance, considering the oxidation mechanism for multi-spot contacts, can be written as,

(3) $$\hat{R}(t,R_{0},t_{m})=\frac{R_{0}}{{\left(1-\sqrt{t/t_{m}}\right)}^{3}\left(1+2\sqrt{t/t_{m}}\right)\left(1+t/t_{m}\right)}$$

*R*_0_ being the initial resistance of the connector, *t* the time elapsed from the installation and *t_m_* the so called maximal lifetime, which corresponds to a vertical asymptote of (3).

**Figure 2:**
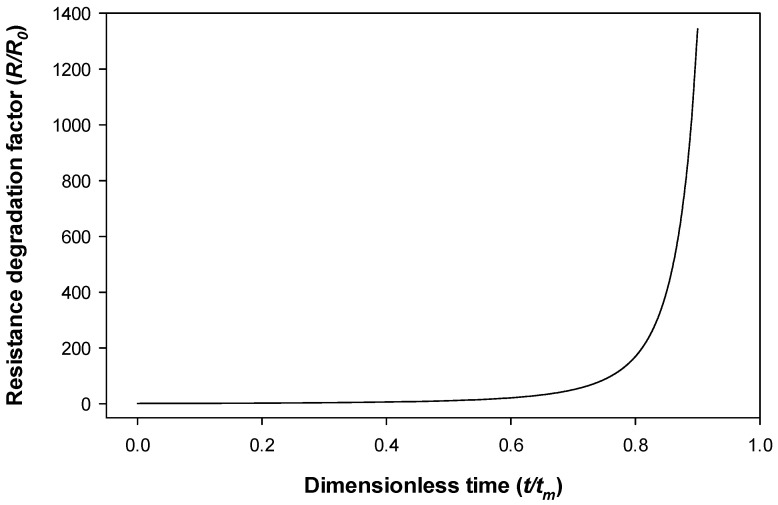
Oxidation multi-spot contact resistance degradation model. As can be observed, at *t = t_m_* the model predicts a vertical asymptote.

Equation (3) applies for multi-spot contacts presenting a beta distribution of the radius of the contact spots [27]. This equation has been chosen since it fits well with the experimental data. Figure 2 displays the time evolution of the resistance according to (3).

### 2.3. Parameter Identification

Once the model in (3) has been established, parameters *R*_0_ and *t_m_* must be identified. To this end, the Nelder-Mead gradient-free minimization algorithm is applied from previous or past data (voltage drop, temperature and current) of the connectors. Therefore, once parameters *R*_0_ and *t_m_* have been identified, by applying (3), which is a simple and fast-to-calculate expression, it is possible to forecast the future values of the connector resistance, which is the basis of the RUL predictive model proposed in this work.

### 2.4. RUL Criterion

**Figure 3:**
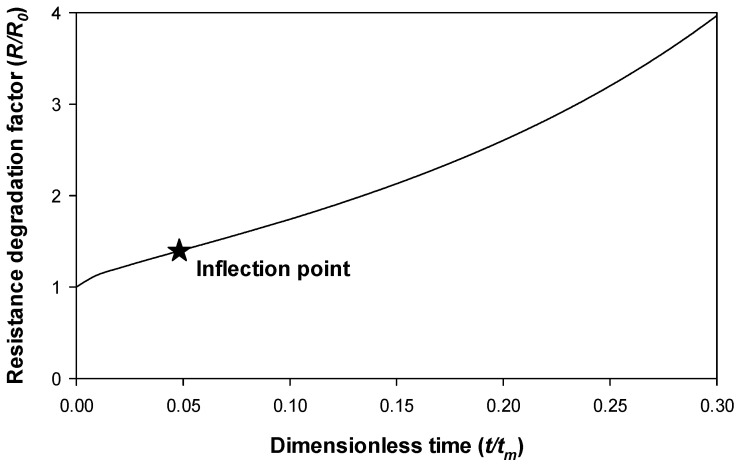
Detail of Figure 2 up to *t/t_m_* = 0.3 together with the proposed RUL criterion, corresponding to the inflection point of (3).

As previously explained, any increase in the resistance of the connector is traduced by more power losses, heat generation and degradation of the contact interface. Therefore, a RUL criterion is proposed based on monitoring of the time evolution of the resistance. In order to generate a robust and simple RUL predictive model, a simple end-of-life criterion is required. To this end it is proposed to determine the inflection point of the resistance degradation curve expressed by means of (3), this being the point at which the resistance transits from convex to concave, i.e., by determining the point in which the increase of the electrical resistance accelerates. After the second derivative of (3) equals zero, the inflection point of (3) occurs at *t* = 0.0482*t_m_*, corresponding to *R* = 1.395*R*_0_, i.e., when the connector’s resistance has increased by at least 39.5% with respect to its initial value *R*_0_, as shown in Figure 3.

The proposed RUL criterion based on the time instant of the inflection point found in Model (3) makes sense, since it is the point from which the derivative of the contact resistance starts to increase. Therefore, above this point, the resistance increases with a consequent higher chance of being quickly degraded.

## 3. Tested Connectors and Experimental Setup to Determine the RUL of the Analyzed Connectors

**Figure 4:**
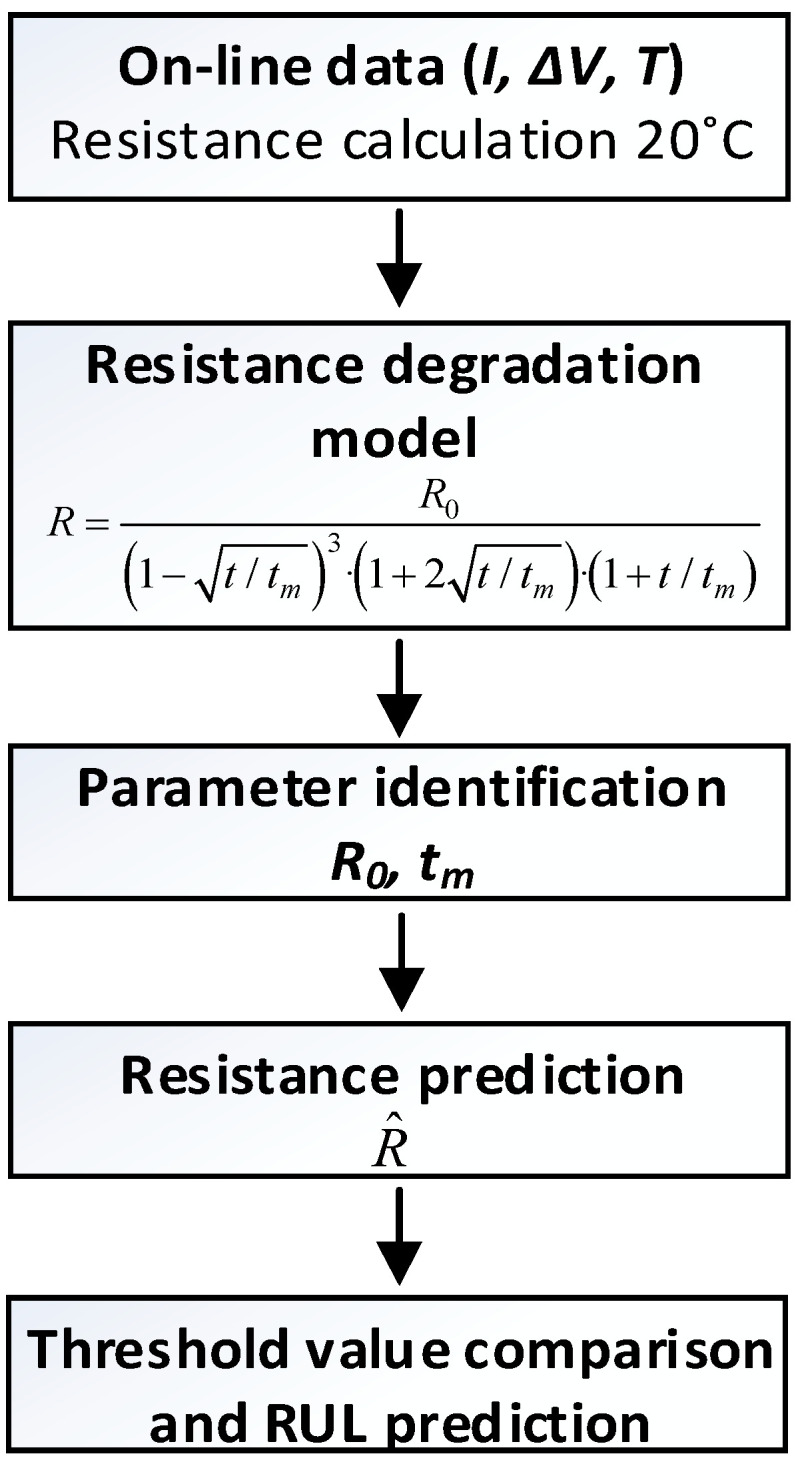
Steps required to determine the RUL of the connectors.

This section presents the method applied in this paper to assess the proposed on-line connector RUL estimation method from the experimental data. Figure 4 summarizes the steps required to determine the RUL of the connectors according to the approach proposed. The first step corresponds to the acquisition of experimental data when the connector is operating under the conditions which will be further specified. The acquired data is the current that flows through the connector, the voltage drop across the connector’s terminals, the phase difference between current and voltage, and finally the working temperature of the connector. According to (2), the resistance is transformed to 20 °C. As mentioned earlier, resistance data is fitted to Model (3) by means of the generalized least squares and Nelder-Mead minimization algorithm. Such procedure allows inference of the model parameters *R*_0_ and *t_m_*, thus predicting the future behavior of the connector’s resistance. From such a prediction, the RUL criterion can be applied and compared against the threshold value to yield the actual RUL estimation.

It is worth noting that the approach proposed in this paper, including the experimental results, are focused of confirming the viability of applying this method to determine the RUL of substation connectors. This implies adding an energy harvesting unit, different sensors (current, voltage drop and temperature) and wireless communications, thus turning the connector into a SmartConnector [33].

### 3.1. Electrical Connectors

**Figure 5:**
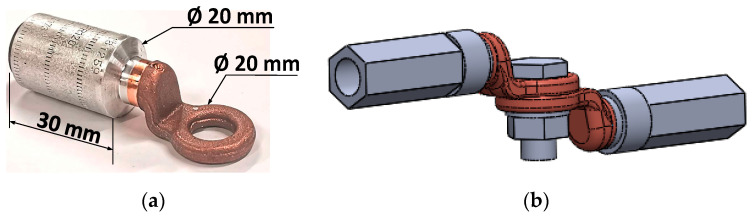
ICAU120 Al-Cu connectors. (**a**) Before compression. (**b**) CAD drawing after compression including the bolting elements.

This section describes the bimetallic friction-welded copper-aluminum ICAU120 Al-Cu compression connectors from the catalogue of SBI Connectors, for aluminum conductors of 120 mm^2^. These are intended for low- and medium-voltage and are shown in Figure 5. The aluminum material is EN AW-1050A aluminum according to the EN 573-3:2014 standard [34], whereas copper material is Cu-ETP according to the EN 13601:2014 standard [35]. To optimize the contact between the connector and the conductor, the barrel is compressed by means of a hexagonal crimping tool (69 MPa BURNDI EP-1HP) [36] and the inner surface of the aluminum barrel is covered with contact grease withstanding 140 °C.

It is noted that the results presented in this work are planned to be applied in substation connectors by means of the SmartConnector project [19]. Apart from the substation connector, the SmartConnector includes a thermal-based energy harvesting system, the sensors unit (temperature, current and voltage drop) and the wireless data transmission system. Therefore, the connectors described in this section serve as a previous stage to validate the feasibility of the proposed approach to estimate the RUL in a faster and economical way since, due to their geometrical dimensions, the current required is lower compared to that required by the substation connectors, thus consuming much less power, whereas the duration of the heat cycle tests is much less.

### 3.2. Connector Degradation Stress by Applying Heat Cycle Tests

In order to acquire on-line experimental data on the degradation of the connectors, they were degraded by applying heat cycle tests according to the IEC 61238-1-3:2018 international standard [37]. Heat cycle tests are commonly used to characterize the thermal behavior of power connectors and to accelerate the thermal ageing process [38]. Heat cycle tests generate thermal expansion and contraction cycles, because of the heating and cooling effects, thus affecting the contact spots at the interface and tending to increase the contact resistance. To this end, an AC (alternating current) electrical current is forced to flow through the loop so that the conductor reaches thermal equilibrium at 120 °C. The IEC 61238-1-3:2018 standard defines the equilibrium as the time instant at which the temperature of the connector and the reference conductor do not change by more than ±2 °C for 15 min. Next, the current is disconnected in order to cool down the loop using forced ventilation, to bring the loop to a temperature ≤35 °C. At this point the next heat cycle starts. A total of 140 heating-cooling cycles according to the requirements of the IEC-61238-1-3:2018 standard were carried out at the AMBER-UPC laboratory. Experimental tests were performed at 20 °C and the temperature, the voltage drop and the current flowing through the connectors were measured during the tests. It is noted that the heat cycles are applied to obtain experimental data to test the suitability of the RUL approach proposed in this paper. In a real application using the SmartConnector, the data from the heat cycle tests will be no longer required since the on-line data provided by the SmartConnector installed in a real substation will be used instead.

**Figure 6:**
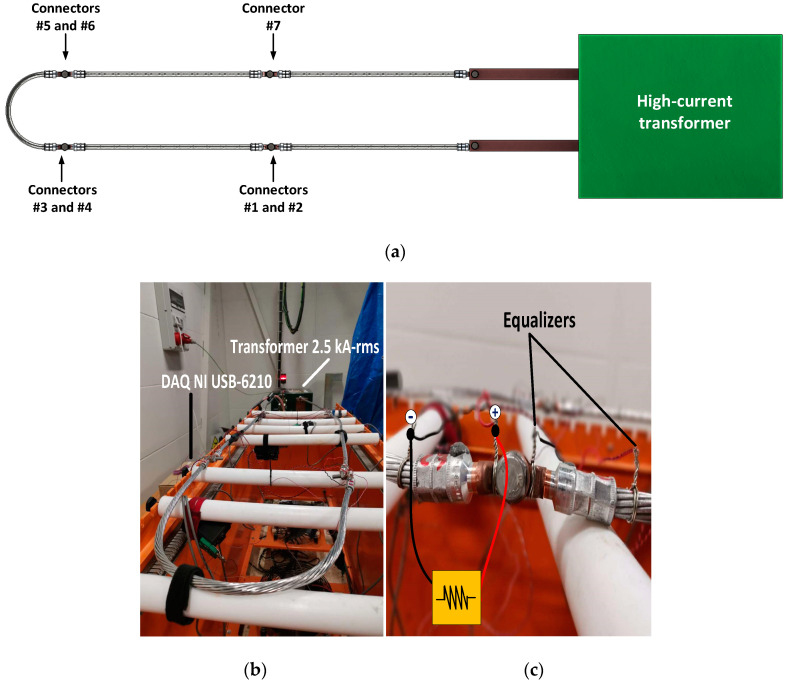
The electrical loop used in the heat cycle tests. (**a**) Schematic of the electrical loop. (**b**) Loop used in the heath cycle tests. (**c**) Measurement of the contact resistance.

The data obtained through the heat cycle tests are used to emulate real service life data. To this end, an electrical loop consisting of seven ICAU120 Al-Cu connectors and an aluminium alloy conductor with a cross-section of 120 mm^2^ was mounted to apply the heat cycle tests, as shown in Figure 6. Wire equalizers were used to equalize the voltage at the measurement terminals and to improve the accuracy of the contact resistance measurement.

Before running the heath cycle tests, the initial DC contact resistance of all connectors was measured by means of the four-wire method in order to obtain a reference value. Next, the heat cycle tests were started so that a total of about 140 heat cycles were completed. Such tests lased about 92.5 h, during which the temperature, current and voltage drop in the seven connectors were acquired every 6 s. To accelerate the degradation process and to reduce the time required to perform the heat cycle tests, the temperature of the reference conductor during the tests was set to 120 °C, which corresponds to an electric current of between 330 A_RMS_ and 380 A_RMS_, but the recommended working temperature is below 90 °C.

The electrical loop during the heat cycle tests was supplied by means of a high-current variable transformer (400 V_RMS_/6 V_RMS_ with a rated output current of 2500 A_RMS_), as shown in Figure 6.

## 4. Sensors and Equipment Used

This section describes the equipment and sensors used to determine the contact resistance, which is the main parameter to determine the performance and the RUL of the connectors.

As explained, before running the heath cycle tests, the initial DC contact resistance of all connectors was measured by means of the four wire method, using a calibrated digital micro-ohmmeter (10–200 A, 0.00 μΩ to 5 Ω, ±0.1%, 0.01 μΩ; model Micro Centurion II from RayTech GmbH, Bremgarten, Switzerland). This is the reference measurement of contact resistance, which is used to check that the connector is properly installed before running the degradation heat cycle tests.

The voltage drop AC waveforms across the terminals of every connector were measured during the heat cycle tests by using a DAQ instrument (absolute accuracy 88 μV, sensitivity 4.8 μV, 16 bits, 250 kSamples/s; model USB-6210 from National Instruments, Austin, TX, USA), which includes eight differential inputs. The 50 Hz waveform of the electric current flowing through the loop was measured with a calibrated Rogowski coil (0.06 mV/A sensitivity, ±0.05% linearity; model CWT500LFxB from Power Electronic Measurement Ltd., Nottingham, UK) connected to the DAQ. The 50 Hz voltage drop and current waveforms were acquired at a rate of 5 kSamples/s. This setup ensures a measuring accuracy of the initial electrical resistance of more than 1 μΩ by applying (2).

The first differential input of the DAQ was used to measure the current, whereas the remaining seven inputs were used for measuring the voltage drop across the connectors. The sample acquisition frequency used in this experiment was six samples/second for the current and voltage measurements, thus allowing calculation of the resistance of every connector once every 6 s. The temperature of the connectors and reference conductor was also measured by using T-type thermocouples and a USB thermocouple data acquisition module (accuracy better than 1 °C, 20 bits, 0.2% ± 0.5 °C accuracy, 0.1 °C sensitivity; TC-08 from Omega, Bienne, Switzerland.

**Figure 7:**
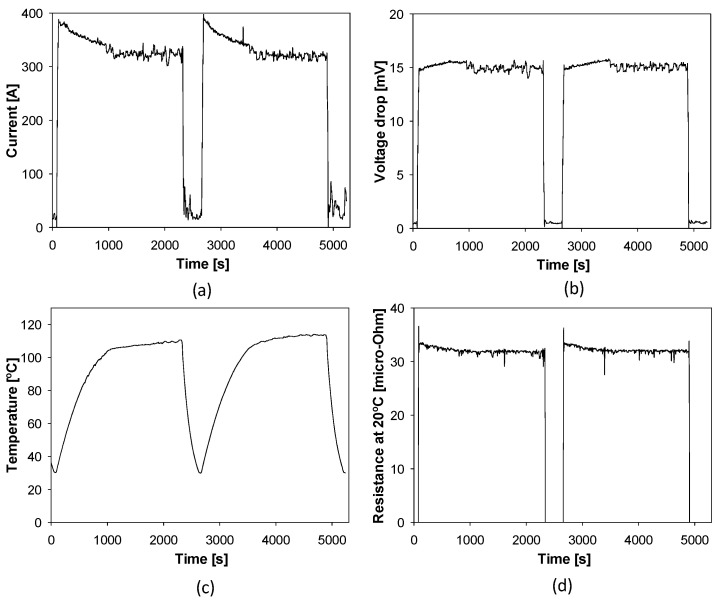
Measurements done in connector #2 during two heat cycles. (**a**) Current. (**b**) Voltage drop. (**c**) Temperature. (**d**) Resistance.

Figure 7 shows the current, voltage drop, temperature and resistance of connector # during two heat cycles.

## 5. Experimental Results and RUL Model Assessment

### 5.1. Experimental Assessment of the Electrical Resistance Degradation Model (ERDM)

**Figure 8:**
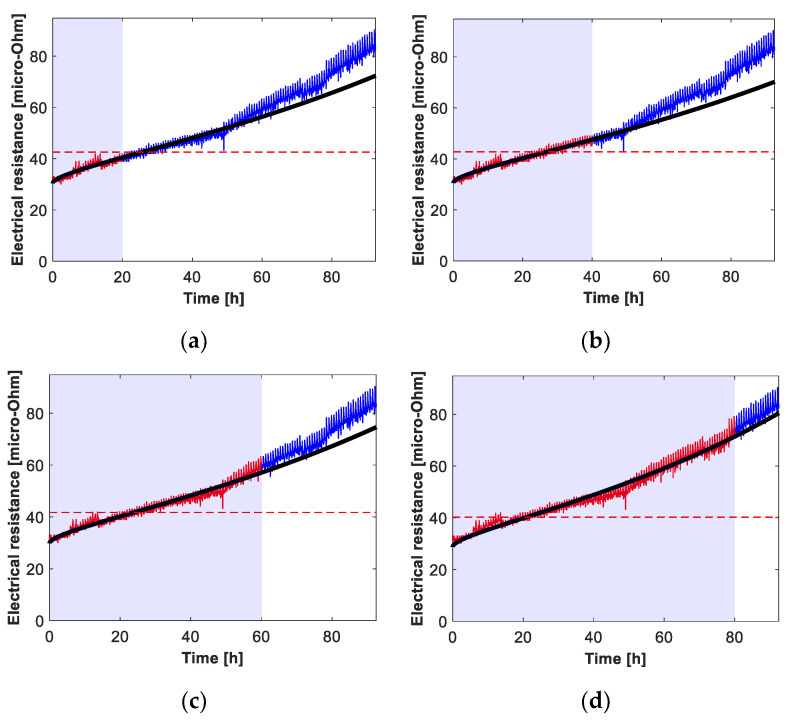
Detail of the fitting of the multi-spot electrical resistance models during the 92.5 h of the heat cycle tests for connector #1. Experimental (red-blue) and fitted (black) values of the electrical resistance versus time and threshold value settled by the inflection point of (3). (**a**) 20–72 model, where 20 refers to the data collected during the first 20 h to fit the model, and 72 refers to the prediction done for the next 72 h (*R*^2^ = 0.874). (**b**) 40–52 model (*R*^2^ = 0.967). (**c**) 60–32 model (*R*^2^ = 0.972). (**d**) 80–12 mode (*R*^2^ = 0.981).

This section assesses the suitability of the multi-spot resistance degradation model described by (3). The full set of experimental values of the electrical resistance of the seven connectors dealt with in this work, obtained from the heat cycle tests, is adjusted by means of (3). As an example, Figure 8 shows the experimental evolution of the connector’s #1 resistance, the fitting values from (3) and the proposed threshold value to determine the RUL. The experimental connector resistance is calculated from the instantaneous electrical current, voltage drop, phase shift and temperature, according to (2). Results presented in Figure 8 show that the heat cycles (heating and cooling cycles) are reflected in the instantaneous values of the resistance, since its profile is not smooth as it contains peaks and valleys corresponding to the heating and cooling phases. Results presented in Figure 8 also show that despite the irregular profile of the resistance evolution with time, the degradation model described by (3) is able to produce a good fitting of the experimental data. To determine the accuracy of the multi-spot model of electrical resistance in predicting the degradation of the connectors, the coefficients of determination *R*^2^ obtained by fitting (3) to the experimental data are summarized in Table 1. Results summarized in Figure 8 and Table 1 indicate the appropriateness and accuracy of the multi-spot resistance degradation model.

### 5.2. On-Line RUL Prediction Based on Different Prediction Horizons

This section describes the experimental results attained through the heat cycle tests and the RUL estimation obtained through the approach shown in Figure 4. The experimental data is split into two sets, i.e., past and future data as in in Figure 8, in order to simulate a real case in which it is supposed to use the current and past values of the connector’s variables (temperature, voltage drop and current), from which the RUL must be determined. The data acquired during the heat cycle tests and labeled as future data are used to assess the performance and accuracy of the RUL model. The data acquired during the first 20 test hours is used to predict the evolution of the connectors’ resistance during the remaining 72 h (20-72), and the same for the first 40 hours to predict the remaining 52 h (40-52), and the 60-32 and 80-12 data models are also analyzed. In a real case, this estimation could be performed on an hourly basis or at whatever suitable interval. Since the data is acquired on-line, the RUL estimation evolves with time according to the contact resistance profile. Sometimes this requires some time before stabilizing, so it is better to have a minimum amount of experimental data collected before applying the RUL prediction, about 20 h in this case.

**Figure 9:**
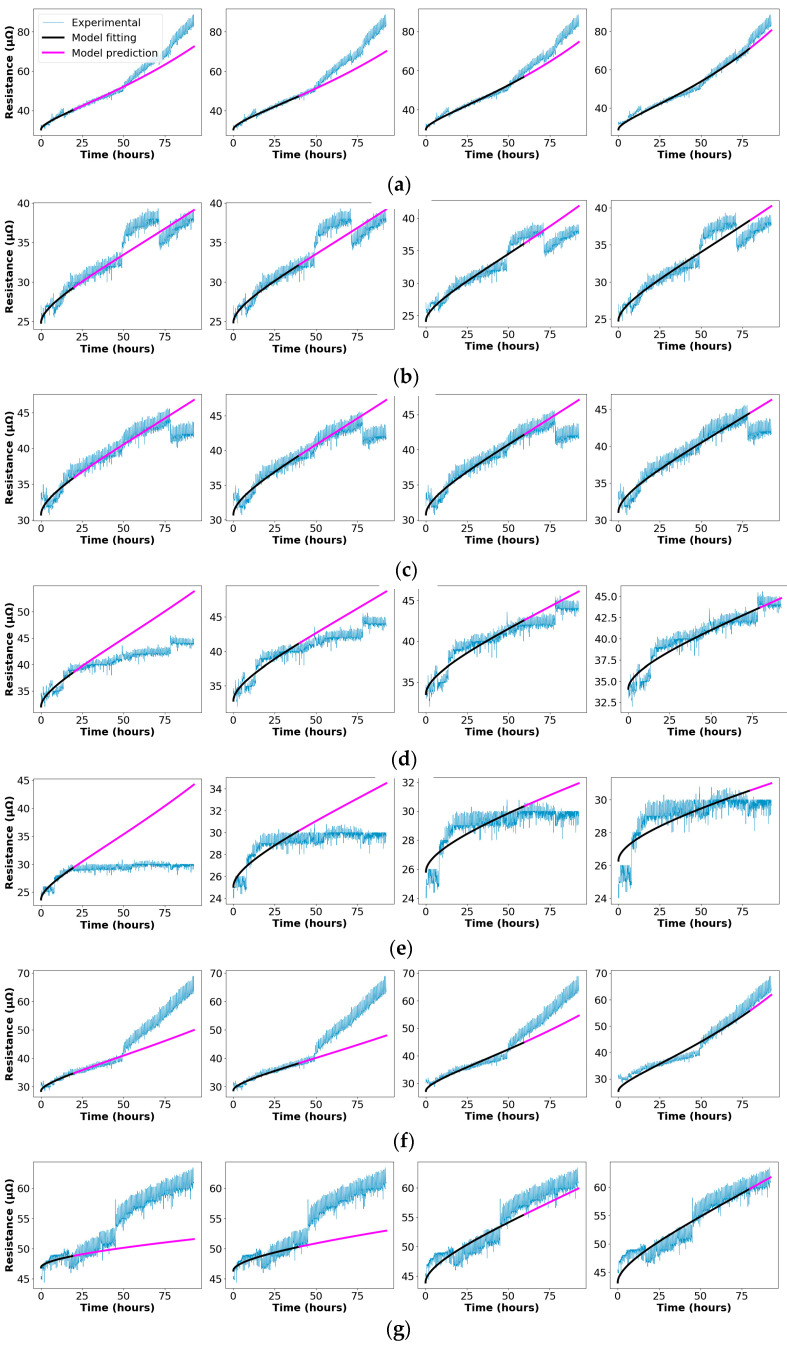
Fitting of the multi-spot electrical resistance models during the 92.5 h of the heat cycle tests until the conductors reach thermal equilibrium at 120 °C for the seven connectors (#1 to #7), considering four models (20-72, 40-52, 60-32 and 80-12 models). (**a**) #1. (**b**) #2. (**c**) #3. (**d**) #4. (**e**) #5. (**f**) #6. (**g**) #7.

Figure 9 shows the RUL estimation for the 20-72, 40-52, 60-320 and 80-12 prediction horizons for all connectors #1 to #7.

Table 2 summarizes the main parameters of the RUL estimation using the already mentioned prediction horizons. The RUL values are referred to time zero (installation time). Results shown in Table 2 clearly indicate that the predictive capability of the proposed model evolves with time, so that the model provides a different RUL value at each time instant.

Results in Table 2 show that each connector has its own RUL evolution profile as a consequence of its particular change in electrical resistance with time. It is important to note that some of the results presented in Table 2 (marked in bold) would not be computed in a real life scenario because the RUL prediction has yielded to an early end-of-life and to the replacement of the connector. See, for instance, connector #1, for which the fitting and RUL estimation carried out at 20 h predicts that it will last only up to hour 25, when it should be replaced.

Table 3 compares the RUL results attained with the electrical resistance degradation model (ERDM) proposed in this work against the widely used autoregressive integrated moving average model, ARIMA (*p,d,q*), where *p* is the AR order, *d* is the differencing order, and *q* is the MA order. Table 3 only analyzes connectors #1-3 and #6 because they reached end of life (EOL) during the experiments. In addition, the ARIMA(2,1,2) model showed better accuracy than other combinations of the (*p,d,q*) orders. Results presented in Table 3 shows the better accuracy of the RUL predictions made by the ERDM proposed in this work, compared with the ARIMA model. It is worth noting that ERDM is appealing because it combines physical knowledge and data driven approaches, whereas ARIMA only relies on a data driven approach. It is noted that data driven approaches usually include three stages, i.e., data acquisition, health index calculation and RUL prediction [39]. In addition, predictions made by ARIMA highly rely on the difficulty in setting a priori the (*p,d,q*) orders. The proposed ERDM based RUL estimation strategy presents a considerably low computational effort, since the average computation time required for fitting and estimating the RUL for each of the 28 calculations summarized in Table 2 is about 5 ms when executed over an Intel Core i7-7700HQ CPU running at 2.8 GHz, with 8 Gb of RAM, whereas ARIMA requires about 1100 ms to perform the same task.

## 6. Conclusions

Power substations are commonly inspected by means of visual checks, using thermal infrared cameras, ultraviolet solar blind cameras or drones equipped with different types of camera. However, these inspection methods cannot be applied very often, since they are expensive and difficult to carry out under adverse weather conditions [40], and do not provide a sufficient amount of numerical data to develop mathematical RUL models, thus this paper contributes in this area. However, the implementation of a SmartConnector in a real high-voltage environment is not fully developed, a challenging task due to the complex systems involved, including energy harvesting, sensing systems and wireless communications. The main drawbacks of this system are related to the use of extra components (the SmartConnector itself) and the extra cost, which can be offset by several advantages, since it allows applying predictive maintenance tasks and to minimize the possibility of sudden faults and the associated costs.

This paper has developed a simple approach with a very low computational burden to determine the remaining useful life (RUL) of power connectors while in the field. An appealing feature of this approach is that it does not require previous degradation tests on the connectors, which consume high amounts of electrical energy, and are expensive and time-consuming. Its main application is found in predictive maintenance plans, since the proposed on-line strategy allows anticipating and planning for the moment in which the connector must be replaced, based on determining the evolution of its RUL via the available data. To this end the voltage and the drop in current flowing across the connector, as well as its temperature, must be acquired on-line and processed to determine the instantaneous value of its electrical resistance. Due to the large number of power connectors installed, their extended service life being longer than that of the electronics required for an on-line monitoring, only some connectors deserve to be instrumented, i.e., those that are in critical links and those that have greater electrical loads, as a poor operating condition could jeopardize the proper operation of critical parts of the system. By using a simple but accurate analytical model of the time evolution of the resistance, i.e., the degradation model, the RUL can be easily predicted. This model depends on two parameters which are identified by fitting the equation describing such a degradation model to the experimental data, by means of a generalized least squares algorithm. The proposed criterion for determining the RUL is based on the inflection point of the equation describing the degradation of the electrical resistance. To validate the proposed approach to determining the RUL, experimental tests performed on seven connectors have been conducted, proving the potential and viability of this method in determining and anticipating when the connector must be replaced before presenting a major failure. It is worth noting that a similar approach can be applied to many other power devices.

## Figures and Tables

**Table 1:** Fitting results of the experimental evolution of the connector’s resistance according to (3).

Connector	#1	#2	#3	#4	#5	#6	#7
*R*_0_ (µΩ)	28.0	25.3	32.0	34.2	24.4	24.9	43.3
*t_m_* (h)	398.2	1281.8	1936.3	2645.7	5582.7	471.7	1754.4
*R* ^2^	0.988	0.895	0.882	0.918	0.893	0.967	0.913

**Table 2:** Fitting parameters and RUL estimation for different connectors using several prediction horizons (time in hours, resistance in µΩ).

Connector		#1	#2	#3	#4	#5	#6	#7
	*R*_0_ (Ω)	30.4	24.8	30.8	32.0	24.9	28.4	46.90
**20-72**	*t_m_* (h)	519.3	1219.8	1369.0	1010.1	11,549.6	905.7	14,306.3
	RUL (h)	25.0	58.8	66.0	48.7	557.1	43.7	690.1
	*R*_0_ (Ω)	**30.6**	24.9	30.8	32.9	24.8	28.6	46.4
**40-52**	*t_m_* (h)	**548.3**	1218.3	1321.1	1502.4	8933.4	1023.7	8144.4
	RUL (h)	**26.4**	58.8	63.7	72.5	430.9	49.4	392.8
	*R*_0_ (Ω)	**29.8**	**24.2**	30.8	33.5	24.8	**27.2**	43.9
**60-32**	*t_m_* (h)	**486.0**	**938.9**	1339.9	2056.2	9408.6	**683.9**	2129.4
	RUL (h)	**23.4**	**45.3**	64.6	99.2	453.8	**33.0**	102.7
	*R*_0_ (Ω)	**28.7**	**24.8**	**31.1**	34.1	24.5	**25.4**	43.2
**80-12**	*t_m_* (h)	**424.6**	**1117.1**	**1481.1**	2595.7	6451.3	**505.5**	1725.6
	RUL (h)	**20.5**	**53.9**	**71.4**	125.2	311.2	**24.4**	83.2

Bold numbers indicate that the connector has reached its RUL.

**Table 3:** RUL estimation for different connectors using ARIMA(2,1,2) and the ERDM proposed in this work.

Connector		#1	#2	#3	#6	Error [h]
	Measured	32.7	53.1	72.2	50.8	-
20-72	ERDM	25.0	58.8	66.0	43.7	26.7
	ARIMA	30.8	54.6	55.6	56.8	26.0
	Measured	32.7	53.1	72.2	50.8	-
40-52	ERDM	-	58.8	63.7	49.4	15.6
	ARIMA	-	82.4	>92	73.1	>51.6
	Measured	-	-	72.2	50.8	-
60-32	ERDM	-	-	64.6	-	7.6
	ARIMA	-	-	83.6	-	11.4
		Total error		ERDM		49.9
				ARIMA		>89.0

## Data Availability

Not applicable.
